# Identification of the cannabinoid receptor 1 antagonist, ibipinabant, as a potent inhibitor of *Neisseria gonorrhoeae*

**DOI:** 10.1128/aac.01231-25

**Published:** 2026-02-04

**Authors:** Autumn S. Dove, Abdallah S. Abdelsattar, Nader S. Abutaleb, Mohamed N. Seleem

**Affiliations:** 1 Department of Biomedical Sciences and Pathobiology, Virginia Polytechnic Institute and State University 1757, Blacksburg, Virginia, USA; 2 Center for One Health Research, Virginia Polytechnic Institute and State University 1757, Blacksburg, Virginia, USA; Columbia University Irving Medical Center, New York, New York, USA

**Keywords:** multidrug-resistant*Neisseria gonorrhoeae*, GPCR inhibitors, antimicrobial resistance, drug repurposing, mouse model of*Neisseria gonorrhoeae *infection

## Abstract

*Neisseria gonorrhoeae,* the causative agent of the second-most prevalent sexually transmitted bacterial disease globally, has been classified as an urgent threat to public health and a high-priority pathogen. Concerningly, *N. gonorrhoeae* has developed resistance to nearly all FDA-approved drugs. Currently, no approved oral therapies exist, with parenteral administration of ceftriaxone as the only available FDA-approved treatment option for multidrug-resistant gonococcal infections. Yet, ceftriaxone-resistant isolates have now been identified globally, further highlighting the urgent need for the development of novel antibacterial agents. In a screen of 2,528 small molecules targeting G-protein-coupled receptors and related signaling pathways, ibipinabant, a potent cannabinoid receptor 1 antagonist, was identified as having the most potent anti-gonococcal activity. Ibipinabant demonstrated potent activity against a panel of 20 *N*. *gonorrhoeae* isolates, without inhibiting some representative *Lactobacillus* species of the vaginal microbiome. A time-kill assay revealed that ibipinabant is bactericidal, clearing the burden of *N. gonorrhoeae* (below the limit of detection) within 12 h. Ibipinabant was also able to clear the intracellular burden of *N. gonorrhoeae* inside human endocervical cells more effectively than the drug of choice, ceftriaxone. This drug was non-toxic against multiple cell lines and did not induce hemolysis of human red blood cells. Finally, in the *in vivo* mouse model of *N. gonorrhoeae* genital tract infection, ibipinabant showed a significant reduction (>95%) in the gonococcal burden after 2 days of treatment. Altogether, these results indicate that ibipinabant is a promising candidate for drug repurposing as a novel antimicrobial against multidrug-resistant *N. gonorrhoeae*.

## INTRODUCTION

*Neisseria gonorrhoeae,* the etiological agent of the sexually transmitted infection (STI) gonorrhea, remains a significant global public health concern, with more than 80 million cases worldwide, 1.5 million of which occur in the United States alone (1–3). *N. gonorrhoeae* can cause a wide range of severe sequelae, leading to complications such as pelvic inflammatory disease, ectopic pregnancy, and infertility in both women and men (4). Additionally, *N. gonorrhoeae* infections can lead to life-threatening complications, including endocarditis, meningitis, and increased susceptibility to human immunodeficiency virus (HIV) or other sexually transmitted diseases (4). Concerningly, *N. gonorrhoeae* has acquired resistance to all currently available antibiotics, leaving an intramuscular injection of ceftriaxone as the only available FDA-approved treatment option, with ceftriaxone-resistant isolates now identified globally (5, 6). Further compounding the problem is that many pharmaceutical companies no longer consider the investment in novel antimicrobial development due to the high cost and risk and low levels of economic return (7–9). Hence, the fear of an era of untreatable gonorrhea calls for an urgent need to develop and discover new therapeutics to treat multidrug-resistant gonococcal infections.

 *De novo* drug discovery is time-consuming and expensive, taking an average of 10 years and potentially costing over a billion dollars (10). One effective alternative strategy for finding new therapeutics is drug repurposing. Drug repurposing enables the use of existing drugs for alternative disease indications, thereby circumventing the arduous process of *de novo* drug discovery (11, 12). In this case, the toxicity and pharmacological properties are more precisely defined than those of newly synthesized compounds (11, 12). This strategy is becoming increasingly successful, with approximately 30% of FDA-approved drugs and vaccines resulting from drug repurposing (13).

 Utilizing a drug repurposing strategy, we screened a library of 2,528 small molecules (MedChemExpress, HY-L006) targeting G-protein-coupled receptors (GPCRs) and related signaling pathways for the ability to inhibit growth of *N. gonorrhoeae*. This library of molecules contained both FDA-approved drugs and compounds that inhibit various GPCR pathway components, as well as drugs in pre-clinical and clinical trials. It has been shown that some bacteria that comprise the gut microbiota can produce small molecules that act as ligands for GPCRs, thus modulating human-microbe interactions (14, 15). Additionally, known GPCR inhibitors have demonstrated antibacterial activity and prevention of intracellular survival of *Coxiella burnettii* (16). Lipopeptides with GPCR-like structures have also shown antibacterial activity against *Escherichia coli* and *Staphylococcus aureus* (17). This led us to test the library of GPCR inhibitors for anti-gonococcal activity. Of these molecules, ibipinabant was identified as having the most potent anti-gonococcal activity. Ibipinabant, also known as SLV-319 and BMS-64656, is a potent cannabinoid receptor 1 (CB_1_) antagonist (18–21). CB_1_ antagonists, including ibipinabant and rimonabant, have been evaluated for obesity and type II diabetes treatments (22). This effect has been associated with changes in the gut microbiota caused by the upregulation of tissue inflammation but not direct antimicrobial activity (23). The CB_1_ antagonists, ibipinabant and rimonabant, were not reported to have antibacterial activity. In this study, we assessed the antibacterial activity of ibipinabant against 20 clinical isolates of multidrug-resistant *N. gonorrhoeae*. We also evaluated its potency in the presence of human and bovine serum albumins. Additionally, we tested this agent against members of healthy vaginal microbiota that can prevent colonization by *N. gonorrhoeae* (24, 25). The *in vitro* cytotoxicity was measured against different cell lines. Further, ibipinabant’s killing kinetics, post-antibiotic effect (PAE), and ability to reduce the burden of intracellular *N. gonorrhoeae* were explored. Finally, the *in vivo* efficacy of ibipinabant was evaluated in a murine model of *N. gonorrhoeae* genital tract infection.

## RESULTS

### Anti-gonococcal activity of ibipinabant

Ibipinabant was identified as the most potent hit in a screen of 2,528 small molecules targeting GPCRs. The antimicrobial activity of ibipinabant was tested against 20 isolates of *N. gonorrhoeae*, including multidrug-resistant strains and 10 WHO reference strains with diverse resistance profiles and known phenotypic and genetic markers (Table S1) . Ibipinabant was found to be potent against all *N. gonorrhoeae* strains tested, with the MICs ranging between 0.03 and 1 µg/mL (Table 1). It inhibited 50% (MIC_50_) and 90% (MIC_90_) of the tested strains at the concentrations of 0.125 and 0.5 µg/mL, respectively. Remarkably, ibipinabant’s activity was comparable to that of the standard-of-care ceftriaxone, which displayed an MIC_50_ of 0.03 µg/mL and an MIC_90_ of 0.25 µg/mL. Additionally, ibipinabant showed lower MICs than azithromycin, which has an MIC_50_ of 0.5 μg/mL and MIC_90_ of 8 μg/mL. The breakpoints for resistance of ceftriaxone and azithromycin are >0.125 and ≥0.5 μg/mL, respectively (26). Furthermore, ibipinabant maintained its activity against azithromycin- and ceftriaxone-resistant strains, although its highest MIC was seen against ceftriaxone-resistant WHO-X as the only exception.

**TABLE 1:** Activity of ibipinabant and control antibiotics (MICs in µg/mL) against *N. gonorrhoeae* isolates^*a*^

*N. gonorrhoeae* strains	Ibipinabant	Ceftriaxone	Azithromycin
WHO-F	0.125	0.004	0.125
WHO-G	0.125	0.004	0.250
WHO-K	0.125	0.063	0.250
WHO-L	0.063	0.250	0.5
WHO-M	0.250	0.016	0.250
WHO-N	0.5	0.004	0.250
WHO-O	0.063	0.031	0.250
WHO-U	0.250	0.002	4
WHO-X	1	2	0.5
WHO-Z	0.125	0.5	1
CDC-174	0.250	0.125	1
CDC-177	0.125	0.016	2
CDC-179	0.125	0.008	8
CDC-181	0.250	0.031	256
CDC-187	1	0.250	2
CDC-197	0.063	0.250	4
CDC-202	0.125	0.008	16
CDC-206	0.125	0.063	0.5
CDC-210	0.125	0.063	0.250
FA1090	0.031	0.002	0.250
MIC_50_	0.13	0.03	0.5
MIC_90_	0.5	0.25	8

^*a*^ MIC_50_: Concentration of test agent that inhibited 50% of the tested strains, MIC_90_: Concentration of test agent that inhibited 90% of the tested strains.

Additionally, in order to investigate whether ibipinabant has activity against other Gram-negative bacteria, we determined its MICs against representative bacterial species such as *Escherichia coli*, *Pseudomonas aeruginosa*, *Acinetobacter baumannii*, *Klebsiella pneumoniae*, and *Salmonella* Typhimurium, and *Staphylococcus aureus* both methicillin-sensitive and resistant strains. The drug was not effective against the tested Gram-negative strains (MICs > 64 μg/mL) (Table 2), but it was effective against some non-*gonorrhoeae Neisseria* species (Table 3). This suggests specific anti-*Neisseria* activity.

**TABLE 2:** MICs (µg/mL) of ibipinabant and control antibiotics against representative Gram-negative and Gram-positive strains

Bacterial strain	MIC (µg/mL)		
	Ibipinabant	Azithromycin	Ceftriaxone
*E. coli* ATCC 2452	>64	16	>64
*P. aeruginosa* ATCC 15442	>64	64	16
*A. baumannii* ATCC 19606	>64	32	32
*K. pneumoniae* ATCC 1706	>64	64	1
*S. pneumoniae* ATCC 51916	>64	>64	16
*S. aureus* NRS384 (USA300)	>64	32	16
MRSA NRS4330	>64	>64	16

**TABLE 3:** MICs (µg/mL) of ibipinabant and control antibiotics against non-gonorrhoeae *Neisseria* strains

*Neisseria* strains	MIC (µg/mL)		
	Ibipinabant	Azithromycin	Ceftriaxone
*N. meningitidis* NR-30542	0.125	0.25	0.004
*N. meningitidis* NR-30536	0.25	0.5	0.004
*N. meningitidis* NR-32113	0.25	1	0.008
*N. meningitidis* NR-32114	0.06	0.5	0.008
*N. meningitidis* NR-32112	0.25	0.5	0.008
*N. flavescens* HM-115	>16	2	0.25
*N. mucosa* HM-242	16	1	0.125
*N. macacae* AR 0951	1	1	0.125
*N. macacae* AR 0952	1	2	0.25
*N. lactamica* AR 0946	4	1	0.125

### Effect of ibipinabant and control antibiotics against representative *Lactobacillus* species of the vaginal microbiota

The vaginal microbiota can interfere with colonization of *N. gonorrhoeae* in the urogenital environment. Therefore, it is preferable for a new anti-gonococcal therapeutic to inhibit *N. gonorrhoeae* while having limited activity toward the normal vaginal microbiota. As such, we tested ibipinabant and azithromycin as a control for antimicrobial activity against representative strains of *Lactobacillus* spp. that comprise the microbiota of the urogenital tract (Table S2). Ibipinabant did not demonstrate an inhibitory effect against any of the *Lactobacillus* strains tested (MICs >128 µg/mL). Contrarily, azithromycin potently inhibited the growth of all *Lactobacillus* strains tested (MICs ≤1 µg/mL) (Table S2).

### Activity of ibipinabant in the presence of serum

A high level of serum binding can affect the potency of a drug *in vivo* by preventing the drug from reaching the site of infection in effective quantities. Thus, we assessed the activity of ibipinabant in the presence of bovine serum albumin (BSA) and human serum albumin (HSA). When exposed to media containing BSA or HSA (4%), the MIC of ibipinabant did not change for *N. gonorrhoeae* WHO-X and increased by only twofold for *N. gonorrhoeae* FA1090 (from 0.031 to 0.063 µg/mL) (Table 4). This trend was seen with control antibiotic azithromycin, which has been shown to have little to no serum binding, while ceftriaxone increased by eightfold for WHO-X (from 2 to 16 µg/mL) and fourfold for FA1090 (from 0.002 to 0.008 µg/mL), which was also expected.

**TABLE 4:** MICs (µg/mL) of ibipinabant against two *N. gonorrhoeae* strains in presence of 4% BSA or HSA

Test agents	*N. gonorrhoeae* WHO-X			*N. gonorrhoeae* FA1090		
	Alone	4% BSA	4% HSA	Alone	4% BSA	4% HSA
Ibipinabant	1	1	1	0.031	0.063	0.063
Ceftriaxone	2	16	8	0.002	0.008	0.008
Azithromycin	0.5	0.5	0.5	0.250	0.5	0.250

### Safety profile of ibipinabant

**Fig 1:**
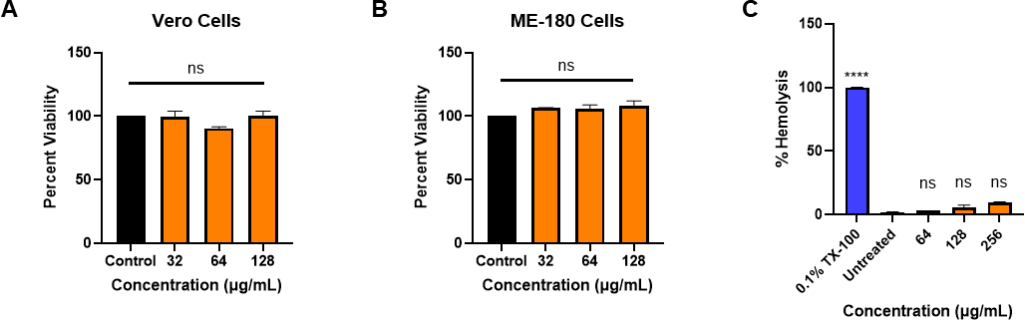
*In vitro* cytotoxicity of ibipinabant against: (**A**) Vero and (**B**) ME-180 cells. Results are shown as a percentage of cell viability relative to the negative control (DMSO). (**C**) Hemolytic activity of ibipinabant against human RBCs. The results are shown as a percentage of RBC hemolysis for each concentration of ibipinabant relative to 0.1% Triton X-100 (TX-100; positive control with complete hemolysis of RBCs). Error bars represent the standard error of the mean; ns, non-significant, ****P < 0.00001.

The cytotoxicity of ibipinabant was initially investigated against two mammalian cell lines, African green monkey kidney epithelial cells (Vero) and human endocervical cells (ME-180). No toxicity was shown to Vero and ME-180 cells. All cells remained viable when exposed to concentrations as high as 128 µg/mL (Fig. 1A and B). The hemolytic activity of ibipinabant was also evaluated using human red blood cells (RBCs). Ibipinabant exhibited almost no lysis of treated human RBCs at concentrations up to 256 µg/mL (Fig. 1C), underscoring human cells’ tolerance to ibipinabant.

### Killing kinetics of ibipinabant against *N. gonorrhoeae*

**Fig 2:**
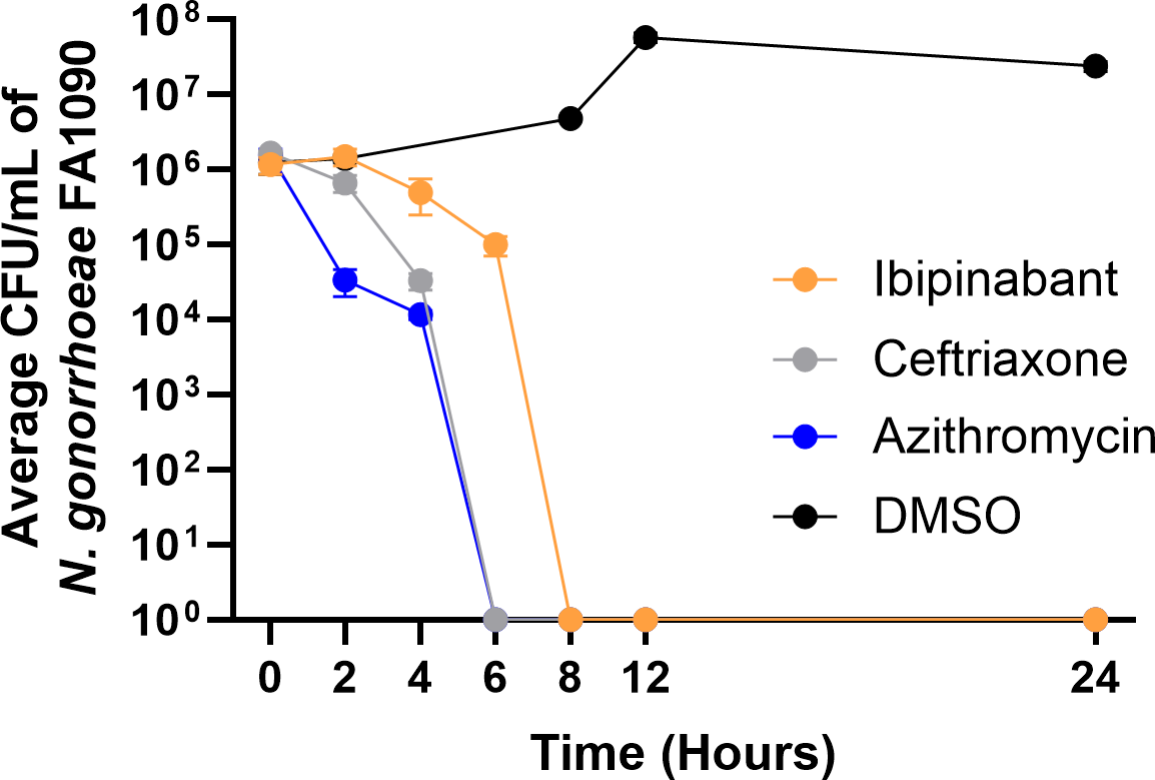
Time-kill assay demonstrating the bactericidal activity of ibipinabant (5× MIC) against *N. gonorrhoeae* FA1090. The data are presented as the mean of log_10_ CFU/mL, and the error bars represent the standard deviation.

A time-kill assay was utilized to determine the mode of killing of ibipinabant against *N. gonorrhoeae.* Ibipinabant (at 5× MIC) exhibited a bactericidal effect against *N. gonorrhoeae* FA1090, reducing the bacterial count by 3 log_10_ CFU/mL after 8 h and completely eradicating the bacterial burden below the limit of detection after 12 h (Fig. 2). The control antibiotic, ceftriaxone, reduced the bacterial count below the limit of detection after 8 h, and azithromycin reduced the bacterial count after 6 h (Fig. 2).

### PAE of ibipinabant against *N. gonorrhoeae*

After confirming the bactericidal activity of ibipinabant against *N. gonorrhoeae*, we examined whether ibipinabant could exhibit a prolonged anti-gonococcal inhibitory activity following a brief exposure period. PAE refers to the period of time that a drug can continue to suppress bacterial growth after a brief exposure. This can aid in the determination of dosing regimens (27). Ibipinabant exhibited a PAE of 8 h against *N. gonorrhoeae* WHO-X (Table S3). Similarly, the control antibiotic azithromycin exhibited a PAE of 8 h.

### Intracellular clearance activity of ibipinabant

**Fig 3:**
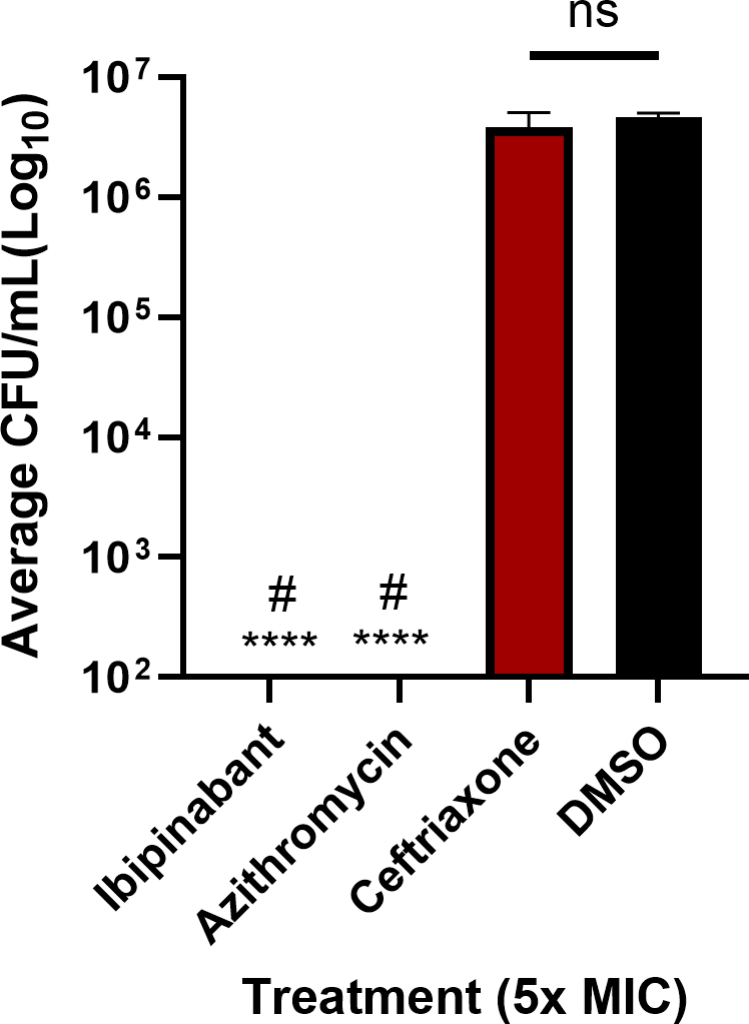
Clearance of intracellular *N. gonorrhoeae* WHO-X harbored in human endocervical cells (ME-180) by ibipinabant compared with control antibiotics, azithromycin, and ceftriaxone (5× MIC). DMSO served as a negative control. Asterisks (*) denote statistically significant differences between test agents and DMSO (untreated) (*P* < 0.05). Pound signs (#) indicate statistically significant differences (*P* < 0.05) between ibipinabant and azithromycin in comparison to ceftriaxone. ns, non-significant.

*N. gonorrhoeae* can invade and replicate inside the mucosal epithelial cell layers in the urogenital tract, resulting in serious infections. As ibipinabant demonstrated potent anti-gonococcal activity against extracellular bacteria, we were interested to explore the ability of ibipinabant to eliminate *N. gonorrhoeae* that has invaded the vaginal endocervical cells. As such, this drug was tested against ME-180 cells infected with *N. gonorrhoeae* WHO-X (ceftriaxone-resistant and azithromycin-sensitive). Using a gentamicin protection assay, we showed that ibipinabant (at 5× MIC) was able to clear the burden of intracellular *N. gonorrhoeae* (below the limit of detection), similar to azithromycin (Fig. 3). However, ceftriaxone showed similar CFUs to the negative control (DMSO).

### *In vivo* efficacy of ibipinabant in a murine gonococcal vaginal infection model

**Fig 4:**
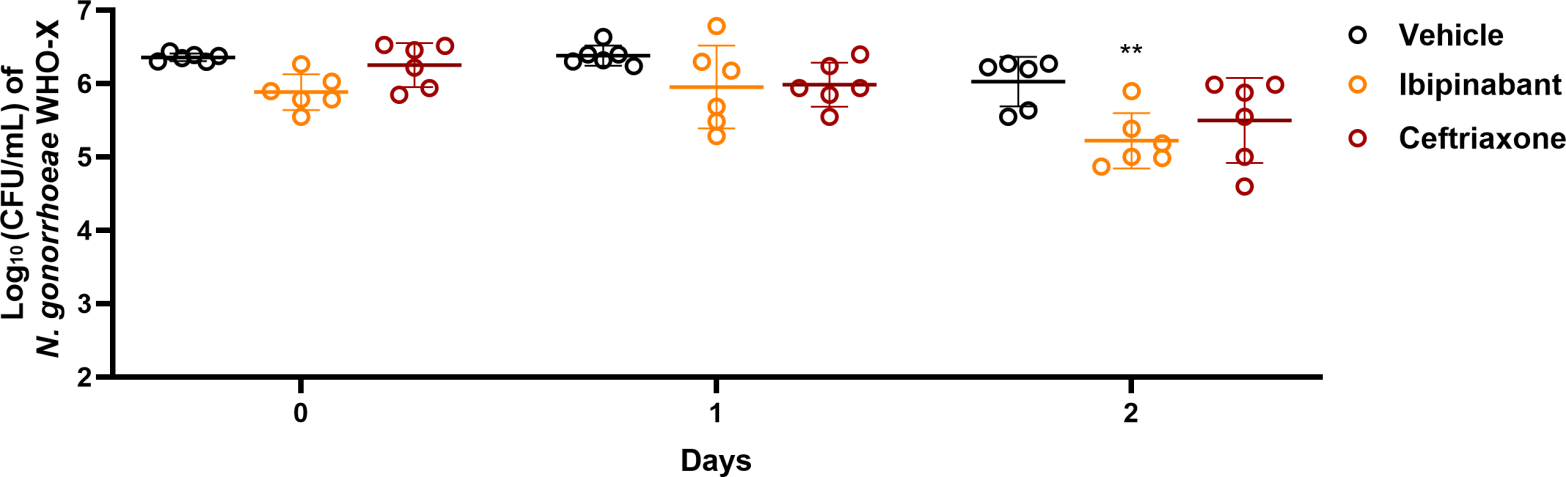
*In vivo* efficacy of ibipinabant in a mouse model of gonococcal infection. Groups of female BALB/c mice (*n* = 6) were inoculated intravaginally at 2 × 10^6^ CFU/mouse of *N. gonorrhoeae* WHO-X. One group was treated with ibipinabant (oral, 20 mg/kg, once daily), one group was treated with ceftriaxone (IP, 15 mg/kg once), and one group was the vehicle control. Significance was analyzed via two-way ANOVA with post-hoc Dunnett’s test for multiple comparisons (**P < 0.01).

Intrigued by the potent activity of ibipinabant against *N. gonorrhoeae in vitro*, we tested the *in vivo* efficacy in the female genital tract gonococcal infection mouse model. In this study, female ovariectomized BALB/c mice were subcutaneously implanted with 5 mg, 21-day controlled-release estradiol pellets 2 days before infection with *N. gonorrhoeae* WHO-X*_rpsL_*_A128G_ (28). This strain is ceftriaxone-resistant and azithromycin-sensitive. We constructed this strain to insert the *rpsl* gene in the genome of *N. gonorrhoeae* WHO-X to induce streptomycin resistance, specifically for use in the *in vivo* mouse model. Mice were treated with ibipinabant (20 mg/kg) orally for two consecutive days. Compared with the vehicle control, mice treated with ibipinabant showed a significant reduction (>95%) in *N. gonorrhoeae* burden after 2 days of treatment (Fig. 4). In contrast, mice treated with a single dose of ceftriaxone (15 mg/kg, intraperitoneal) did not exhibit a significant reduction in *N. gonorrhoeae* burden (Fig. 4).

## DISCUSSION

The high incidence of gonococcal infections and the skyrocketing development of antibiotic resistance pose significant public health challenges. It was reported that at least nine countries show elevated levels of isolates displaying resistance to the current drug of choice, ceftriaxone (ranging from 5% to 40%) (29, 30). Several combined factors have led to the incidence and difficulty of treatment of gonococcal infections. For instance, various stages in the infection process, mucosal adherence, invasion of mucosae, localized inflammatory response, and systemic dissemination can affect therapeutic targeting of this pathogen (31, 32). Moreover, *N. gonorrhoeae* has a high-frequency antigenic and phase variation of surface adhesins in addition to suppressing effectors and regulators of the immune system, allowing this pathogen to evade the immune system, which increases the difficulty of treatment (32–34). Presently, no effective vaccines have been developed to prevent infection by *N. gonorrhoeae*. Additionally, the lack of development of novel antimicrobials and the rising development of resistance toward the currently used antibiotics have created critical concern. As such, the development of novel antimicrobials that can target *N. gonorrhoeae* is of paramount necessity.

Drug repurposing is a strategy that has emerged to circumvent the lengthy and expensive traditional *de novo* drug discovery process. We utilized this strategy to identify novel candidates for treating multidrug-resistant *N. gonorrhoeae* infections.

Ibipinabant was identified in a screen of 2,528 small molecules targeting GPCRs and associated signaling pathways as having potent *in vitro* anti-gonococcal activity. Ibipinabant is a CB_1_ inhibitor that has been explored along with other CB_1_ inhibitors for the treatment of obesity, type II diabetes, and decreasing food and alcohol overconsumption (18–21, 35–39). CB_1_ is a class A GPCR possessing the canonical seven transmembrane domains (7TM). GPCRs have almost exclusively been discovered and studied in eukaryotes. However, a recent study reported eukaryotic GPCRs as possible descendants of prokaryotic sodium-translocating rhodopsins (40). Interestingly, CB_1_ is a Class A GPCR from the mammalian rhodopsin superfamily (41). Therefore, we hypothesized that *N. gonorrhoeae* may have GPCR-related receptors and pathways (particularly related to CB_1_). Comprehensive elucidation of the target(s) of ibipinabant in *N. gonorrhoeae* will be the subject of future investigation.

In the present study, ibipinabant was evaluated against a panel of multidrug-resistant *N. gonorrhoeae* clinical isolates. Ibipinabant potently inhibited *N. gonorrhoeae* growth with MICs from 0.03 to 1 µg/mL. Ibipinabant’s MIC_50_ and MIC_90_ were comparable to those of ceftriaxone, and it exhibited potent activity against the ceftriaxone-resistant strains. Moreover, ibipinabant was more effective than azithromycin, maintaining potent activity against azithromycin-resistant strains. No antibacterial activity was demonstrated against representative members of Gram-negative or Gram-positive bacteria, suggesting a potential selective activity against *Neisseria* species.

The dysbiosis of the urogenital tract enhances the gonococcal colonization and infection. A healthy microbiome can provide many benefits to prevent colonization and the establishment of infections and can aid in fighting off an ongoing infection. Not only can the vaginal microbiota outcompete *N. gonorrhoeae* for attachment to the urinary tract, but they also aid in creating an acidic environment that can also inhibit *N. gonorrhoeae* colonization (42–44). Broad-spectrum antimicrobials, including the current first-line treatment options (ceftriaxone and azithromycin) for gonococcal infections, are known to disrupt the healthy microbiome, which can lead to negative post-treatment outcomes (45, 46). Identifying therapies that can protect healthy microbiota while maintaining selectivity for *N. gonorrhoeae* can help achieve more favorable clinical results. Therefore, we assessed ibipinabant’s activity against representative members of the normal human vaginal microbiota. Ibipinabant was shown to have no effect on the *Lactobacillus* spp. tested (MICs >128 µg/mL). In contrast, azithromycin, in agreement with previous reports, exhibited potent inhibitory activity against these microbiota strains (43, 47, 48). These results demonstrate a potential advantage for ibipinabant as an anti-gonococcal therapeutic to selectively inhibit the pathogenic gonococci without disrupting the beneficial vaginal microbiota.

We next assessed the killing kinetics of ibipinabant. Ibipinabant exhibited bactericidal activity, eradicating the high starting inoculum of *N. gonorrhoeae* below the limit of detection within 12 h. This bactericidal activity is an advantage for ibipinabant as a potential therapeutic for *N. gonorrhoeae,* as drugs with bactericidal activity have several advantages, including limiting the spread of infection, potentially reducing the emergence of bacterial resistance, and shortening the duration of treatment, which is very important as compliance is a concern for such multidrug-resistant STIs (49).

 For the determination of effective dosing regimens, finding the PAE is an essential step. Longer PAEs have been associated with longer dosing intervals, which is important for patient compliance, especially for STIs. Additionally, longer dosing intervals can contribute to lowering the cost of these drugs for patients due to fewer necessary doses (27). PAE studies performed after bacterial exposure to 5× MIC for 1 h found that ibipinabant was able to suppress the growth of *N. gonorrhoeae* for 8 h post-exposure, equivalent to the control drug azithromycin.

 *N. gonorrhoeae* can invade epithelial cells and survive intracellularly, which can lead to disseminated and persistent infections, infection of the uterus and fallopian tubes, and eventually infertility (50–52). The standard-of-care ceftriaxone does not effectively clear intracellular gonococcal infections, due to multiple reasons, including its complex and bulky structure, and its high hydrophilicity and low active transport (53–56). However, drugs like azithromycin can penetrate cells. As such, new therapeutics with intracellular activity are important. Ibipinabant and azithromycin were able to clear *N. gonorrhoeae* burden inside ME-180 cells below the limit of detection, whereas ceftriaxone was ineffective. The results indicate that ibipinabant can presumably enter endocervical cells at a concentration that will significantly reduce intracellular *N. gonorrhoeae* at a rate superior to the current drug of choice, ceftriaxone.

 Finally, the promising features of ibipinabant prompted us to evaluate its *in vivo* efficacy in a murine vaginal infection model against *N. gonorrhoeae* WHO-X (ceftriaxone-resistant). We previously established this mouse model using a streptomycin-resistant strain of *N. gonorrhoeae* WHO-X developed by our group (28). Ibipinabant was able to lower the CFU counts of *N. gonorrhoeae* in mice by 1 log_10_ CFU/mL after 2 days of oral treatment. This was significantly more effective than the drug of choice, ceftriaxone. Consequently, ibipinabant has strong potential for clinical success as a therapeutic against multidrug-resistant *N. gonorrhoeae*.

To conclude, in this study, we have reported the selective bactericidal activity of ibipinabant against *N. gonorrhoeae in vitro* and *in vivo*. Our results suggest that ibipinabant has potential as a novel anti-gonococcal agent. There are opportunities for further optimization of this compound to develop more potent and safe analogs for use against *N. gonorrhoeae* in future studies. Further investigation of ibipinabant as a novel anti-gonococcal agent is warranted to clearly elucidate the antibacterial mechanism of action.

## MATERIALS AND METHODS

### Bacterial strains, chemicals, and media

Isolates of *N. gonorrhoeae* and representative Gram-negative strains were received from the U.S. Centers for Disease Control and Prevention (CDC) and the American Type Culture Collection (ATCC), and *Lactobacillus* strains were obtained from the Biodefense and Emerging Infections Research Resources Repository (BEI Resources).

Chemicals and media were purchased commercially: GC agar base, Chocolate II agar, dried bovine hemoglobin, Brucella broth, and IsoVitaleX (Becton, Dickinson, and Company, Cockeysville, MD), heart infusion agar (Hardy Diagnostics, Santa Maria, CA), yeast extract and dextrose (Fisher Bioreagents, Fair Lawn, NJ), hematin, pyridoxal, and nicotinamide adenine dinucleotide (NAD) (Chem-Impex International, Wood Dale, IL), protease peptone and VCNT supplement (Oxoid, Lenexa, KS), phosphate-buffered saline (PBS) (Corning, Manassas, VA), ceftriaxone and azithromycin (TCI America, Portland, OR), ibipinabant (TargetMol, Boston, MA), Tween 80 (Acros Organics, Fair Lawn, NJ), estradiol pellets (5-mg, 21-day controlled-release) (Innovative Research of America, Sarasota, FL), and Dacron swabs (Medical Packaging Corporation, Camarillo, CA).

### Screen of a library of 2,528 small molecules that target GPCRs for antibacterial activity

A bacterial dilution was diluted in Brucella broth supplemented with yeast extract, dextrose, proteose-peptone, NAD, pyridoxal, hematin, and Isovitalix to a McFarland of 1.0. The library of 2,528 small molecules (MedChemExpress, HY-L006) targeting GPCRs and related signaling pathways was diluted using this broth to a final concentration of 1 µg/mL in 96-well plates. Plates were incubated overnight at 37°C and 5% CO_2_. The inhibition of growth by the compounds was determined visually.

### MIC determination against *N. gonorrhoeae* strains

The MIC values of ibipinabant, azithromycin, and ceftriaxone against *N. gonorrhoeae* strains were determined (57–63). Information on the *N. gonorrhoeae* strains can be found in Table S1. Briefly, a bacterial dilution (McFarland 1.0) was prepared and diluted in supplemented Brucella broth to achieve a bacterial concentration of 1 × 10^6^ CFU/mL. Diluted bacteria were incubated with varying concentrations of ibipinabant, azithromycin, or ceftriaxone at 37°C and 5% CO_2_ for 24 h. MICs were determined visually.

### Effect of ibipinabant against representative vaginal microbiota

Antimicrobial susceptibility testing against representative members of *Lactobacillus* spp. comprising the normal human vaginal microbiota was conducted using the broth microdilution as defined in previous reports (62, 64–68). Lactobacilli were cultured on MRS agar for 48 h at 37 °C in the presence of 5% CO_2_. MRS broth was used to dilute a 0.5 McFarland standard of *Lactobacillus* species to an approximate concentration of 5 × 10^5^ CFU/mL and incubated with serial dilutions of ibipinabant or azithromycin before visual determination of MIC values.

### *In vitro* toxicity

The *in vitro* cytotoxicity for ibipinabant was assessed using human cervical cells (ATCC HTB-33, ME-180) or African green monkey kidney cells (ATCC CCL-81, Vero), as described elsewhere (65, 69–73). Cells were seeded in 96-well tissue culture-treated plates at a density of 1 × 10^5^, followed by an overnight incubation at 37°C and 5% CO_2_ in a humidified environment. Ibipinabant or the equivalent DMSO was serially diluted in McCoy’s 5A medium (ME-180) or DMEM (Vero) with 10% fetal bovine serum (FBS, USA Scientific, Inc.). PBS was used to wash cells three times, then cells were incubated with the diluted compounds or the DMSO control for 24 h. Following the incubation, cells were washed with PBS three times, then the assay reagent MTS 3-(4,5-dimethylthiazol-2-yl)−5-(3-carboxymethoxyphenyl)−2-(4-sulfophenyl)−2*H*-tetrazolium) (Promega, Madison, WI, USA) was added. Reduction of the dye was measured in a Tecan plate reader (OD_490_). The measure of viable cells after treatment with each compound was expressed as a percentage of the DMSO control.

Ibipinabant’s hemolytic activity was evaluated as previously described (74, 75). Single-donor human RBCs (Innovative Research, MI, USA) were suspended in PBS at a concentration of 4% v/v. Ibipinabant was diluted in PBS to 64, 128, and 256 µg/mL and incubated with 4% RBCs for 1 h at 37°C. Centrifugation (800 × *g* for 10 min) preceded the reading of the supernatant in a Tecan plate reader (OD_540_) to assess hemolysis. Triton X-100 was used as a positive control to represent total hemolysis.

### Time-kill assay

A time-kill assay was performed, as described previously, against *N. gonorrhoeae* FA1090 to determine whether ibipinabant is bacteriostatic or bactericidal *in vitro* (58, 76–78). *N. gonorrhoeae* was grown to logarithmic phase using Brucella broth and further diluted to reach an inoculum of 5×10^6^ CFU/mL. Ibipinabant, azithromycin, ceftriaxone, or DMSO was then added at 5× MIC in triplicate. A volume from each sample was serially diluted and plated onto chocolate II agar plates at times 0, 2, 4, 6, 8, 12, and 24 h. Plates were incubated overnight at 37°C and 5% CO_2_ to determine the CFU count.

### Post-antibiotic effect of ibipinabant against *N. gonorrhoeae*

The PAE for ibipinabant and azithromycin was determined using a method described in previous studies (64, 79–82). Briefly, bacteria were grown in supplemented Brucella broth to logarithmic phase, followed by dilution to approximately 1 × 10^6^ CFU/mL. Test agents were added (5× MIC), then incubated for 1 h at 37°C and 5% CO_2_. After treatment, samples were diluted 1:500 in a fresh Brucella broth to diminish the drug concentrations and further incubated at 37°C and 5% CO_2_ for 12 h. Samples were collected from each group every 2 h, serially diluted in PBS, and plated onto chocolate II agar plates. Plates were incubated overnight at 37°C and 5% CO_2_ to determine viable CFUs. The PAE was calculated using the following equation: T-C, where T is the time required for bacterial culture treated with the drug to increase by one log_10_ after removal of the drug, and C is the time required for the negative control to increase by one log_10_.

### Intracellular clearance assay

An intracellular bacterial clearance experiment was utilized to investigate the ability of ibipinabant to enter human cervical cells and reduce the burden of intracellular *N. gonorrhoeae*, as described in other studies (50, 51, 83). Briefly, human cervical cells were seeded in 96-well tissue culture-treated plates (∼1 × 10^5^ cells per well) for 24 h at 37°C with 5% CO_2_ in a humidified environment. Cells were maintained in McCoy’s 5A medium supplemented with 10% FBS. Following incubation, the cells were washed three times with PBS and infected with *N. gonorrhoeae* strains WHO-X at a multiplicity of infection of 1:100 for 6 h in a humidified environment at 37°C with 5% CO_2_. The cells were washed three times with PBS containing 320 μg/mL gentamicin and further incubated for 2 h with gentamicin (320 μg/mL) to eliminate and remove extracellular bacteria. ME-180 cells were then exposed to ibipinabant, azithromycin, ceftriaxone, or the equivalent DMSO at 5× MIC, and incubated for 24 h at 37°C with 5% CO_2_ in a humidified environment. After incubation, cells were washed with PBS and lysed using 0.01% Triton X-100 to collect intracellular bacteria. The lysate was serially diluted in PBS and plated on chocolate II agar plates. Plates were incubated at 37°C with 5% CO_2_ for 24 h. Experiments were performed using six samples for each treatment group, and the experiment was repeated twice.

### Evaluating the *in vivo* efficacy of ibipinabant in the mouse model *N. gonorrhoeae* genital tract infection

** **Mice were housed in individually ventilated cages and received food and water *ad libitum* throughout the experiment. The mouse model for *N. gonorrhoeae* infection was performed, as previously described (28, 82, 84–86). The mice used were ovariectomized 8-week-old female BALB/c mice (Jackson Laboratory, Bar Harbor, ME). On Day −2, mice were implanted subcutaneously with a 5 mg 21-day-released estradiol pellet using stainless steel precision trocars (Innovative Research of America, Sarasota, FL), followed by a drop of tissue adhesive (3M Animal Care Products, Saint Paul, MN) to seal the injury.

Antibiotics were administered to increase susceptibility to *N. gonorrhoeae* by limiting commensal bacteria. Mice were injected intraperitoneally with 0.6 mg of vancomycin and 1.2 mg of streptomycin on Days −2 to +1. The drinking water was replaced on Day −2 with sterilized water containing 0.4 g/L trimethoprim. Trimethoprim water was renewed every other day, including the addition of streptomycin sulfate (2.4 g/L) starting on Day +2 until the end of the experiment.

On Day 0, the vagina of each mouse was inoculated intravaginally with 20 µL of 2.24 × 10^6^ CFU/mouse of *N. gonorrhoeae* WHO-X (streptomycin-resistant) (28). On Day +2, mice were randomly allocated into groups (*n* = 6) and administered ibipinabant (20 mg/kg) or the vehicle orally for 2 days. One group of mice was administered a single dose of ceftriaxone (15 mg/kg, water, intraperitoneal) as a control.

 Vaginal swabs were collected daily through gentle insertion of a moistened Dacron swab into the vagina of anesthetized mice, followed by suspension of the swab in 100 µL of Brucella broth containing 0.05% saponin. Samples were serially diluted and plated onto GC agar supplemented with vancomycin, nystatin, and trimethoprim (85). Plates were incubated overnight at 37°C and 5% CO_2_, then enumerated for CFUs. A small aliquot of the sample was also cultured on heart infusion agar to observe the presence of commensal microbiota. Gram staining was performed to further identify the commensals (if any). The presence of enteric Gram-negative rods could prevent *N. gonorrhoeae* from colonization, giving a false-positive result. As such, any mice colonized with enteric Gram-negative rods were excluded from the study. Mice were humanely euthanized via carbon dioxide asphyxiation at the conclusion of the experiment.

### Statistical analysis

Student’s *t*-test, one-way analysis of variance (ANOVA), and two-way ANOVA with post=hoc Dunnett’s test for multiple comparisons were used to determine statistical significance. The data were considered statistically significant when *P* < 0.05. Statistical significance was indicated by asterisks (*P < 0.05, **P < 0.01, ***P < 0.001, ****P < 0.0001). Error bars represent the standard error of the mean (SEM). All statistical analyses were performed using GraphPad Prism version 10.4.0 for Windows (GraphPad Software Inc., La Jolla, CA).

## SUPPLEMENTAL MATERIAL

Supplemental material aac.01231-25-s0001.docx Tables S1 to S3.

ASM does not own the copyrights to Supplemental Material that may be linked to, or accessed through, an article. The authors have granted ASM a non-exclusive, world-wide license to publish the Supplemental Material files. Please contact the corresponding author directly for reuse.
