# Supplementary material for: Identification of the cannabinoid receptor 1 antagonist, ibipinabant, as a potent inhibitor of Neisseria gonorrhoeae
Source: Antimicrob Agents Chemother. 2026 Feb 4;70(3):e01231-25. doi: 10.1128/aac.01231-25 (PMC12959157; doi:10.1128/aac.01231-25)
Supplement: Supplemental material — Tables S1 to S3. [file aac.01231-25-s0001.docx]

**Table S1. The resistance profile for 20 multidrug-resistant *N. gonorrhoeae* strains.**

| ***N. gonorrhoeae* strains** | **Resistance Profile** |
| --- | --- |
| **WHO-G** | Ciprofloxacin, tetracycline |
| **WHO-K** | Ciprofloxacin, tetracycline, cefixime, penicillin |
| **WHO-L** | Ciprofloxacin, tetracycline, azithromycin, ceftriaxone, penicillin |
| **WHO-M** | Ciprofloxacin, tetracycline, penicillin |
| **WHO-N** | Ciprofloxacin, tetracycline, penicillin |
| **WHO-O** | Tetracycline, spectinomycin, penicillin |
| **WHO-U** | Tetracycline, azithromycin, penicillin |
| **WHO-X** | Tetracycline, ciprofloxacin, penicillin, ceftriaxone, cefixime |
| **WHO-X*rpsL*A128G** | Streptomycin, tetracycline, ciprofloxacin, penicillin, ceftriaxone, cefixime |
| **WHO-Z** | Tetracycline, ciprofloxacin, penicillin, ceftriaxone, cefixime |
| **CDC-174** | Tetracycline, penicillin, ciprofloxacin |
| **CDC-177** | Tetracycline |
| **CDC-179** | Azithromycin |
| **CDC-181** | Tetracycline and azithromycin |
| **CDC-187** | Ciprofloxacin, penicillin |
| **CDC-197** | Ciprofloxacin, penicillin |
| **CDC-202** | Azithromycin |
| **CDC-206** | Tetracycline, ciprofloxacin, penicillin |
| **CDC-210** | Ciprofloxacin, penicillin |
| **FA1090** | Streptomycin |

**Table S2.** MICs (µg/mL) of ibipinabant and the control antibiotic azithromycin against vaginal microbiota isolates.

| ***Lactobacillus* strains** | **Ibipinabant** | **Azithromycin** |
| --- | --- | --- |
| ***L. gasseri* HM-642** | >128 | ≤1 |
| ***L. jensenii* HM-639** | >128 | ≤1 |
| ***L. jensenii* HM-105** | >128 | ≤1 |
| ***L. johnsonii* HM-643** | >128 | ≤1 |
| ***L. crispatus* HM-638** | >128 | ≤1 |

**Table S3.** Post-antibiotic effect (PAE) of ibipinabant against *N. gonorrhoeae* WHO-X

| **Bacterial Strain** | **PAE** | |
| --- | --- | --- |
|  | **Ibipinabant** | **Azithromycin** |
| ***N. gonorrhoeae***  **WHO-X** | 8 | 8 |
